# Energy saving, load bearing and attachment mechanism on ice and frozen ground of biomimetic mechanical foot

**DOI:** 10.1371/journal.pone.0296689

**Published:** 2024-01-26

**Authors:** Guoyu Li, Rui Zhang, Hao Pang, Yexuan Luo, Yong Hong, Zhisong Li, Hua Zhang, Lige Wen

**Affiliations:** 1 School of Mechanical Engineering, Shanghai Dianji University, Shanghai, People’s Republic of China; 2 Key Laboratory of Bionic Engineering, Ministry of Education, Jilin University, Changchun, People’s Republic of China; 3 Aerospace System Engineering Shanghai, Shanghai, People’s Republic of China; 4 School of Mechanical and Aerospace Engineering, Jilin University, Changchun, People’s Republic of China; Ningbo University, CHINA

## Abstract

The frozen ground robot can be widely and prospectively applied in plentiful fields, such as military rescue and planet exploration. Based on the energy-saving, load-bearing, and attachment functions of reindeer hooves, we studied the kinematics of reindeer feet and designed a biomimetic energy-saving attachment mechanical foot (mechanical foot I) and two contrast mechanical feet (mechanical feet II and III). The energy-saving and load-bearing performances of the biomimetic mechanical foot were tested on a motion mechanics platform, which revealed this mechanical foot was adaptive to three types of ground (frozen ground, ice, and water ice lunar soil). Mechanical foot I possesses the functions of elastic energy storage and power consumption reduction, and its power range is from -2.77 to -27.85 W. Compared with mechanical foot III, the load-bearing ability of mechanical foot I was improved by the dewclaws, and the peak forces in the X, Y, and Z directions increased by about 2.54, 1.25 and 1.31 times, respectively. When mechanical foot I acted with more- smooth surface, the joint range of motion (ROM) increased, changes of the three-directional force at the foot junction decreased. The forces were the lowest on ice among the three types of ground, the X-, Y- and Z-directional changes were about 62.96, 83.7, and 319.85 N respectively, and the ROMs for the ankle joint and metatarsophalangeal joint of mechanical foot I were about 17.93° and 16.10°, respectively. This study revealed the active adaptation mechanism between the biomimetic mechanical foot and ice or frozen ground, and thus theoretically underlies research on the biomimetic mechanical foot.

## 1. Introduction

With the development of artificial intelligence, robots have gradually entered daily life and replaced humans in production and manufacturing. For instance, Atlas and Digit robots can be applied into logistics and package delivery [1,2]. The unmanned military equipment has become an inevitable trend, and various military robots are increasingly applied. For example, Packbot robots can complete reconnaissance, exploration, and explosive device handling [3]. As the only component in contact with the ground, the mechanical foot is of great significance in improving the trafficability and mobility of robots on extreme ground conditions [4].

Biomimetic foot robots have high mobility and environmental adaptability on unconventional surfaces by using discrete point contacts and flexible limb structures, and thus are applied into exploration and transportation in high-risk environments [5]. BigDog, a military quadruped robot developed by Boston Dynamics, is equipped with advanced controllers (e.g., terrain sensors and dynamic control) to coordinate leg movements, ensuring motion balance and stability. Its controllers adapt to terrain changes through terrain measurement and attitude control, which enable the robot to move in various outdoor terrains, such as snow, ice, and frozen ground [6]. The MIT analyzed the dynamics, energy consumption, and speed of quadruped movement and thereby designed a lightweight bionic mechanical foot based on the cheetah hindlimb of tendon bone collaborative positioning. This mechanical foot can reduce foot mass while meeting stiffness and strength, avoid the generation of large bending moments, and facilitate efficient energy use and high-speed operation [7]. With breakthroughs in lightness and balance, the second-generation Atlas is allowed by the internal sensors and LiDAR positioning to avoid obstacles, maintain balance, and complete indoor, outdoor, and snow movements. However, when walking in snow, the robot still slips and can only collect pose data through sensors to adjust its motion state and maintain balance [8]. Thanks to precise control algorithms, image measurement, and gait adjustment, the footed robot possesses strong anti-interference ability, and can initially walk on frozen soil to certain extent [9]. However, the poor walking stability and relatively simple foot structures of robots result in little research on the bionics of the mechanical foot on frozen ground.

Frozen ground, as an extreme ground, is distributed worldwide. The total area of permafrost in the world is roughly 35 million Km^2^, accounting for 25% of the total area of the Earth. In recent decades, scientists have discovered water ice on the moon through continuous lunar exploration, but are still studying the specific location and content of water ice [10]. The NASA and ESA explored lunar water ice in the permanent shadow areas of the North and South Poles by employing lunar probes, such as Clementine, Lunar Prospector, and SMART-1. Preliminary conclusions were drawn that water ice may exist [11–13]. In 2008, the LCROSS and LRO cooperated to impact the moon. Thermal imaging, near-infrared spectroscopy, ultraviolet spectroscopy and other technologies were used to identify water vapor in the impact sputtering, which proved the existence of water ice on the moon [14]. In 2010, the Mini-star radar, developed by NASA and carried on Luna I in India, detected more than 40 craters containing water ice in the north lunar pole, with an estimated content of ~ 600 million tons of water ice [15]. Moon water ice is not only a hot topic for international exploration, but also a strategic resource for the future. Currently, various world aerospace powers have formulated plans to explore and sample lunar water ice. The European Space Agency proposed a plan to build a lunar village. China planned for multiple explorations and sampling of lunar water ice before 2030 [16]. Lunar water ice mainly exists in permanently shaded areas (craters), which wheeled robots can hardly pass through. Nevertheless, owing to excellent motion performance and environmental adaptability, footed robots have good application prospects in this aspect [17].

Under the laws of natural selection, animals have evolved their foot structures, movement, and functions to adapt to their environment [18]. The engineering application on special structures of animal feet based on biomimetic methods has long been a source of problem-solving and inspiration for humans [19]. Mechanical feet, designed based on a hoofed animal as a biomimetic prototype, have excellent adaptability and motion characteristics in various ground movements. Abad designed a goat hoof-like mechanical foot with the characteristics of flexible joints, strong attachment ability, cushioning, and vibration reduction. The front end of the hoof and the convex crown pattern improve attachment performance by embedding them into the ground and enlarging the contact area, respectively [20]. Horses have excellent athletic characteristics (e.g., mobility, load-bearing capacity, and endurance) in their feet during exercise [21]. Garcia et al. designed a bionic mechanical foot based on effective leg length, kinematics, and foot mass distribution by simulating the horse muscle system and connecting elastic elements and excitation units, which improved the agility of the mechanical foot [22]. They further proposed key factors that affected its agility [22].

As a typical polar migratory animal belonging to Cervidae, Artiodactyla, reindeer (*Rangifer Tartandus*) exhibit various excellent characteristics in their hooves [23]. Reindeer own a hoof structure suitable for migration in complex environments [19]. Reindeer can adapt to various ground environments, such as permafrost [24]. In particular, the hooves of reindeer are highly trafficable on the frozen surface, and their hoof-shell structure and plantar fur play an anti-slip role and can enlarge the contact area with the ground when walking on frozen ground [25]. Reindeer migrate over long distances on land seasonally, and some reindeer populations migrate farther than other land mammals [26].

Based on the excellent characteristics of reindeer hooves (e.g., energy conservation, load-bearing, and attachment), we designed a biomimetic mechanical foot, and thus provided researchers with new design mentality in terms of mechanical foot cushioning, energy conservation, and attachment. On the basis of the interaction between the mechanical foot and frozen ground in combination with kinematic mechanics analysis, we analyzed the mechanism of the biomimetic mechanical foot on ice and frozen ground. This study provides new mentality and references for the research and application of military robots, civilian robots, and walking platforms into deep space exploration at lunar poles.

## 2. Mechanism of reindeer hind-foot movement

### 2.1 Samples

**Fig 1:**
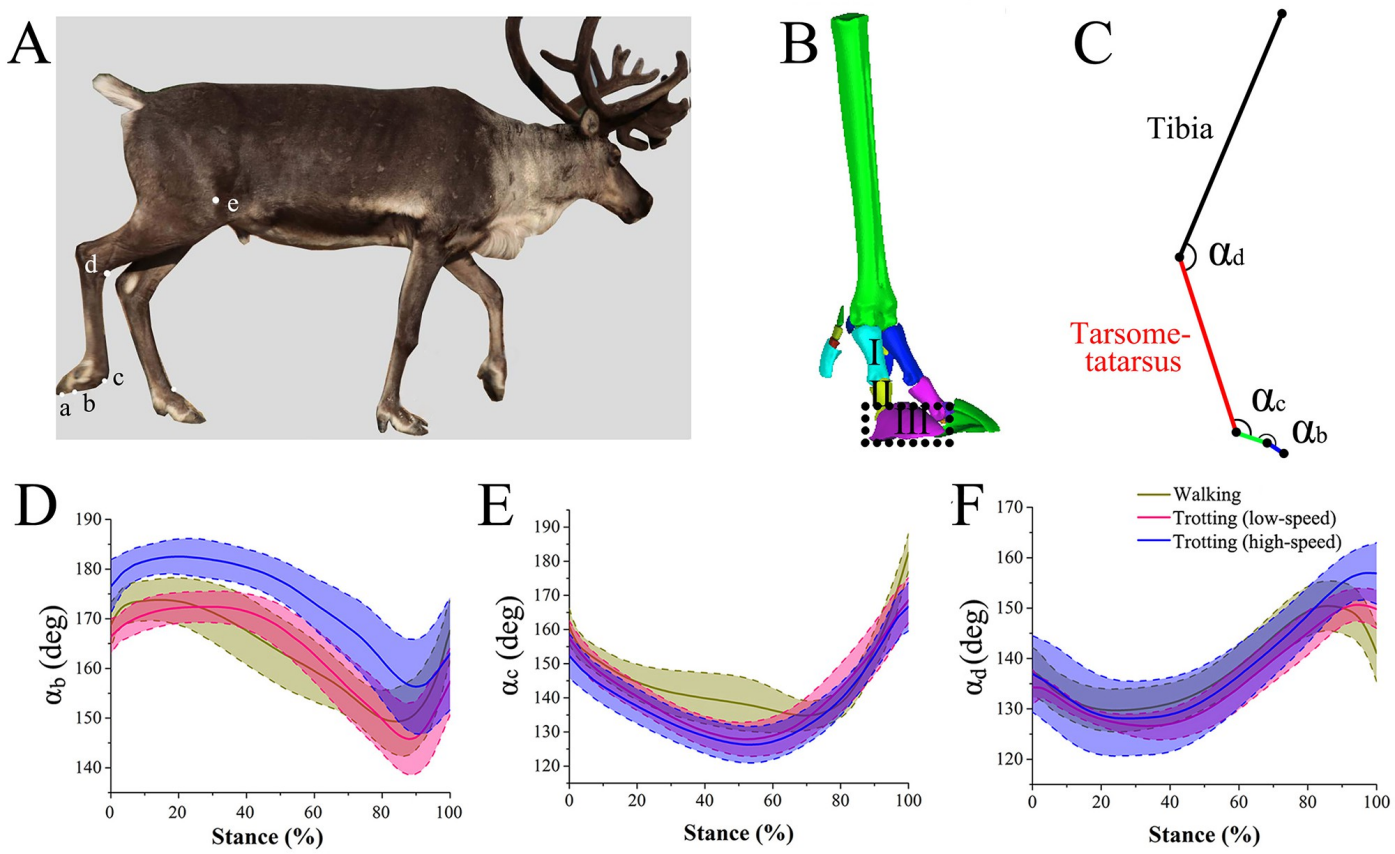
Changing curves of joint angle in right hind limbs of reindeer. A. Mark-point positions on right hind limbs (*a*, *b*, *c*, *d*, and *e*). B. A 3D Model of the foot based on CT scans of the right hind limb of adult male reindeer. C. Main joint angles of hind limbs (*α*_b_, *α*_c_ and *α*_d_). D, E, F: Changes in *α*_*b*_, *α*_*c*_ and *α*_*d*_ during the stance phase. Definition: I, II, III: First, middle and third digits respectively. Yellow, pink, and blue lines represent walking, slow trotting, and fast trotting, respectively. Solid and dotted lines represent mean and standard deviation, respectively.

We selected 7 male eight-year-old adult reindeer from the Ewenki breeding farm in Genhe City, China. The masses, shoulder widths, and body lengths of the four tested samples were 118.75 ± 14.93 kg, 1.22 ± 0.51 m, and 1.89 ± 0.83 m, respectively (mean ± standard deviation). The samples were in good physical conditions and had not undergone any surgical treatment or other invasive procedures. The reindeer were enclosed in outdoor guardrails without restrictions to food or water (the guardrail area was about 2×103 m^2^, close to a semi-wild state), and were released to the wild mountains and forests at night. The biological samples of reindeer are shown in Fig 1A. Extracts from two reindeer that died of natural causes were CT-scanned via reverse engineering. A series of processing steps were performed in GeomagicStudio, such as mesh healing, relaxation, smoothing, and 3D modeling of reindeer hind limbs (Fig 1B). The main joint angles of the hind limbs were shown in Fig 1C.

### 2.2 Kinematics

The data of the four samples were combined to obtain the mean and variance of the joint angles *α*_b_, *α*_c_ and *α*_d_ (Fig 1D–1F respectively) during walking, slow trotting, and fast trotting in the stance phase. The changing trends of the joint angles in the hind limbs during the stance phase are similar among different movements. Specifically, *α*_b_ decreased (dorsiflexion) in the early stage (0% -60%) of the stance phase, and increased (plantarflexion) in the later stage (60% -100%). During all movements, the joint range of motion (ROM) significantly differed among the three joint angles of the hind limbs. The ROM of *α*_c_ was the highest, and was about 47.69°, 40.68°, and 40.37° in walking, slow trotting, and fast trotting, respectively. The ROM of *α*_c_ decreased with the increasing movement speed, because the toe flexion of the metatarsophalangeal (MTP) joint was large when a hind leg was about to leave the ground during walking. The minimum ROMs of the ankle joints of hind limbs (*α*_d_) during walking, slow trotting, and fast trotting were about 20.73°, 24.05°, and 28.87º, respectively. The ROMs of the interphalangeal joint (*α*_b_) at these phases were about 24.47º, 26.60º, and 26.18°, respectively. Hence, the hind limb joint angles of reindeer can strongly adapt to different movement gaits and speeds.

## 3. Design and processing of the biomimetic mechanical foot

### 3.1 Structure of the mechanical foot

**Fig 2:**
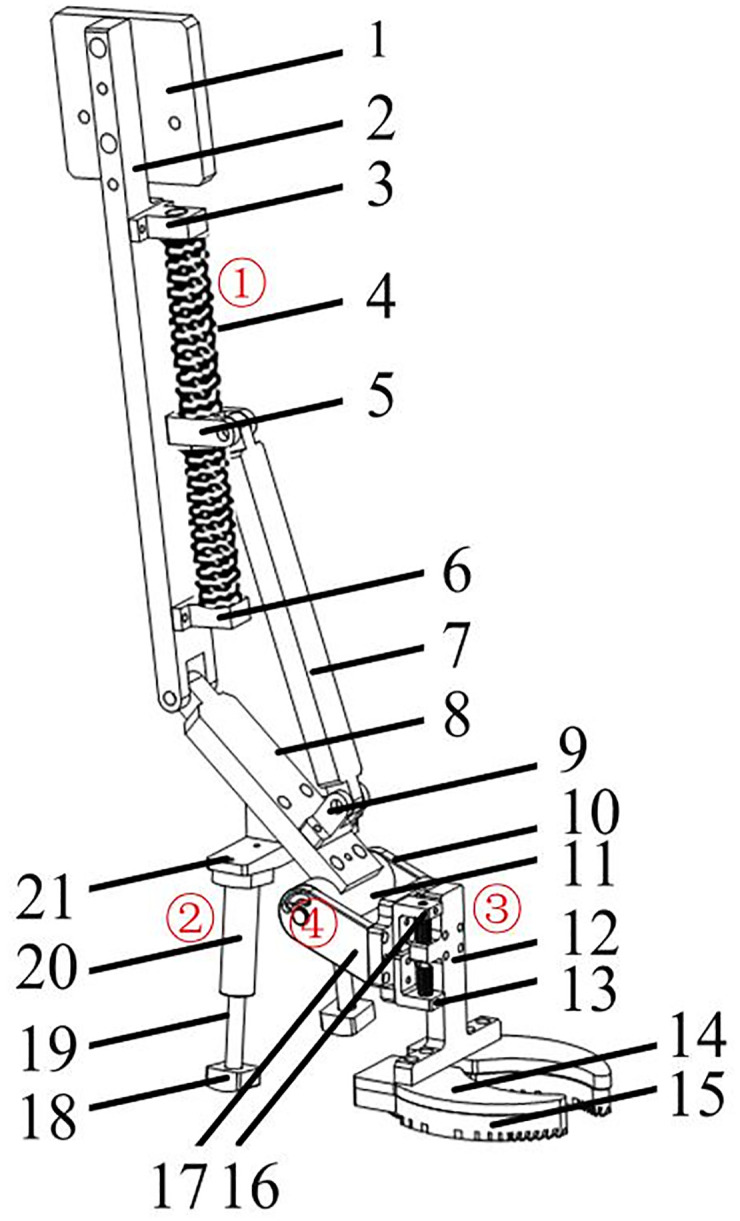
Multi-functional biomimetic mechanical foot. 1—Motor connection plate, 2—Fixed connection plate, 3—Upper fixed support plate, 4—Slide rail, 5—Movable block, 6—Lower fixed support plate, 7—Connection plate, 8—Movable plate, 9—Movable support plate, 10—Fixed plate of torsion spring, 11—Connection column, 12—Slider connection plate, 13—Lower adjustment- column connecting plate, 14—Foot-end connection block, 15—Load-bearing toe, 16—Upper section column connection plate, 17—Side fixed plate, 18—Support block, 19—Telescopic column, 20—Spring-fixing sleeve, 21—Fixing plate of buffering support, ①, ②, ③, ④—First, second, third, and torsion springs respectively.

The structure of the kinematic mechanical foot was designed according to the motion trajectories of the MTP joint and the ankle joint, hoof joint ROM, and the proportion and size of the hoof structure (Fig 2). Based on the energy storage and release characteristics of hoof joints, utilization of elastic components in the MTP joint and ankle joint of the mechanical foot is conducive to energy saving of the bionic foot. A simulated dewclaw structure that was beneficial for improving the buffering effect of the foot was added to the bionic mechanical foot. The length, width, and height of this bionic mechanical foot were about 460, 150, and 800 mm, respectively.

### 3.2 Biomimetic mechanical foot processing

**Fig 3:**
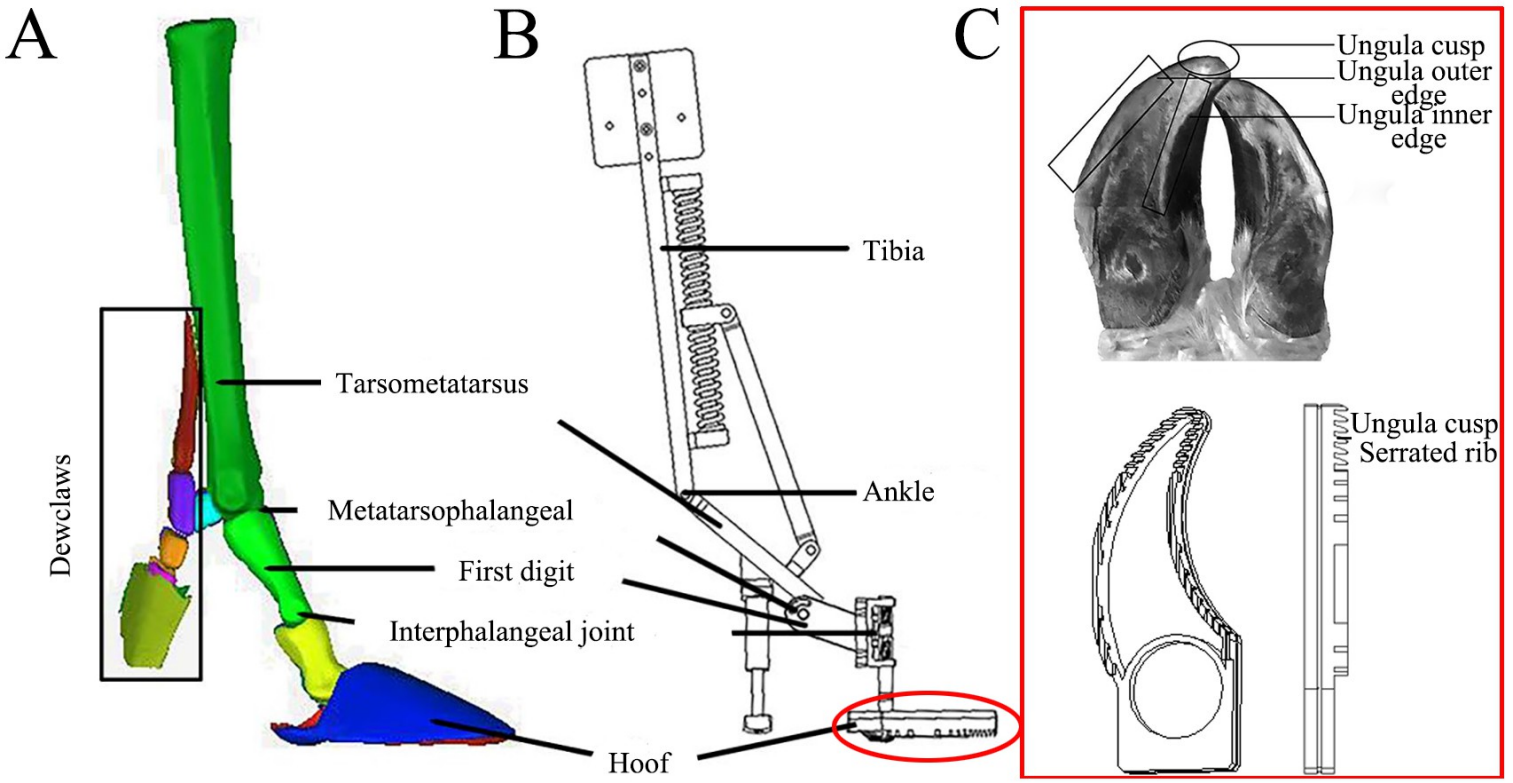
Comparison of the foot structure of the bionic mechanical foot with the biological prototype. (A) Reindeer hoof skeleton model, (B) Structure of the biomimetic mechanical foot, (C) Design of biomimetic foot end.

The bionic mechanical foot structure and the biological prototype were compared in Fig 3A and 3B. The fixed connection plate (2), upper fixed support plate (3), slide rail (4), movable block (5), and lower fixed support plate (6) simulate the function of the reindeer ankle joint, and play a role in storage and propulsion. To achieve the motion state of the mechanical foot from ground touching to ground leaving, we shall combine and design the stiffness coefficients of the first, second, third, and torsion springs. The stiffness coefficients of the first, second, and third springs are about 4.4, 6.5, and 17.8 N/mm, respectively, and their wire diameter, outer diameter, and length are 1.8, 28.0, 130.0 mm; 2.0, 14.0, 80.0 mm; 2.5, 10.0, 20.0 mm, respectively. The side fixed plate (17) and torsion spring fixed plate (10) simulate the first segment of the phalanx, and serve to connect the movable plate (8) and the foot ends (12, 13, 14, and 15). The movable block (5) simulates the energy storage and release functions of the ankle joint, and plays a role in connecting the slide rail (4) and the connecting plate (7). The support block (18), telescopic column (19), spring-fixing sleeve (20), second spring, and fixing plate of buffering support (21) form a mechanical structure resembling a suspension shoe, and play a role in auxiliary support and buffering. When the biomimetic mechanical foot touches the ground, the angles of the movable plate (8) with the fixed connection plate (2) and the side fixed plate (17) are about 134.17 and 153.68°, respectively, which simulate the angles of the biological ankle joint and the MTP joint respectively. As the main landing component of the foot, the load-bearing toe (15) provides the main support function, while the dewclaw plays an auxiliary support role, which are beneficial for further stabilizing the motion of the bionic mechanical foot. When the bionic mechanical foot leaves the ground, the spring releases the stored energy and converts it into kinetic energy, reducing energy consumption and improving the efficiency of foot movement. The load-bearing toe (15) is a biomimetic foot end with ridges and convex crowns selected through foot end design and attachment testing (Fig 3C).

## 4. Motion mechanics of the biomimetic mechanical foot

### 4.1 Energy conservation

#### 4.1.1 3D force measuring device for mechanical foot

**Fig 4:**
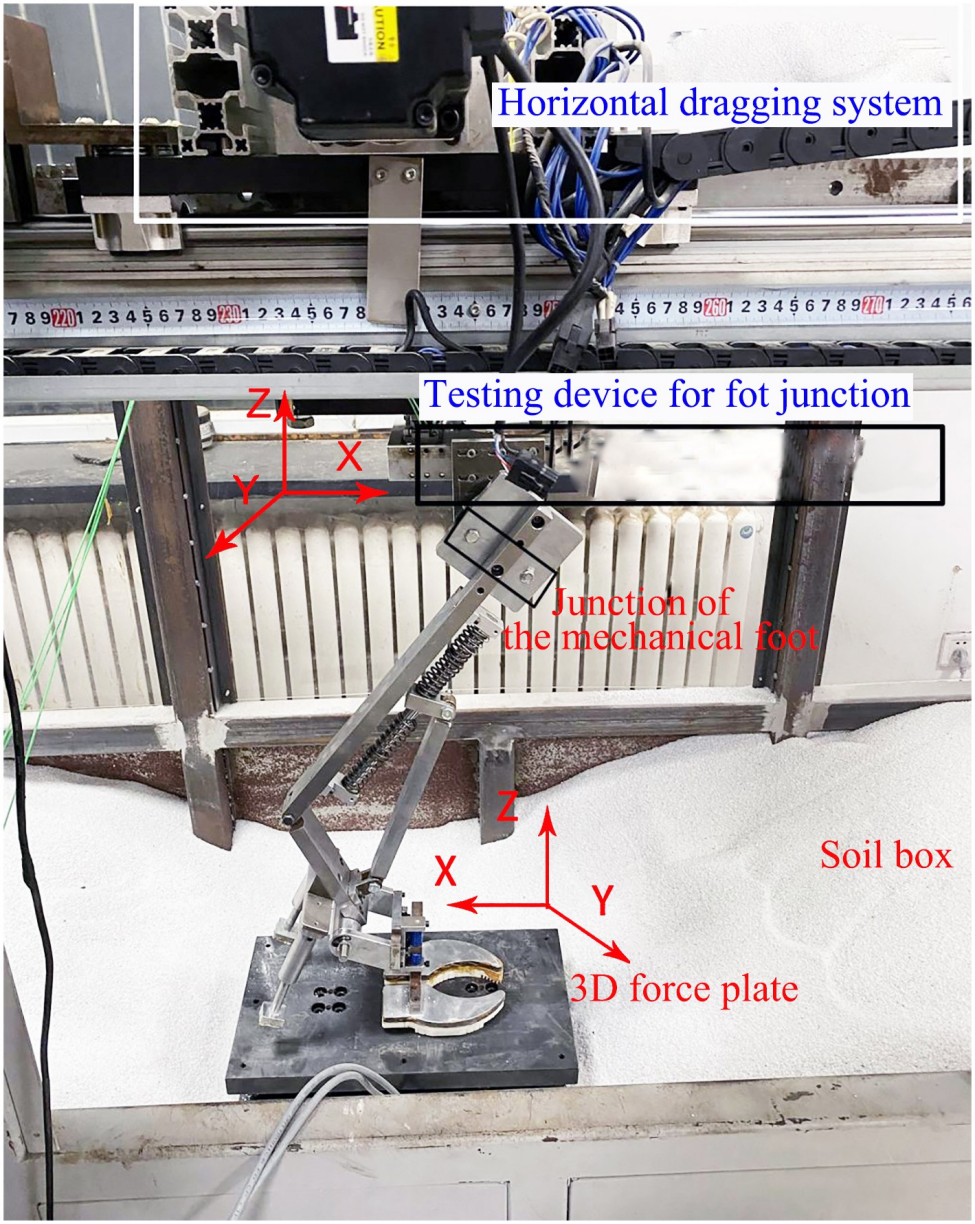
Biomimetic mechanical foot kinematics platform and the 3D force platform.

The kinematic and mechanical properties of the mechanical foot were tested on basis of the biomimetic mechanical foot motion mechanics platform and a 3D force platform (Shenzhen Neten). The foot motion mechanics platform consists of a soil tank, a horizontal drag system, a control computer, and a testing system (Fig 4). Quartz sand was utilized on soil grooves at a certain height and compacted. The upper end of the foot was connected to the 3D force testing system of the motion mechanics platform, so the foot can rotate axially along the testing system. The 3D force testing platform was used to measure the 3D force of the foot sole. The biomimetic mechanical foot was installed on the motion mechanics platform equipped with a three-way force sensor, and was driven by the motor to move horizontally forward, thus achieving the stance action of the foot. Meanwhile, the collection frequency of the foot motion mechanics platform was 50 Hz. The 3D force platform was used to test the plantar pressure of the mechanical foot during stance phase, and its collection frequency, maximum range, and accuracy were 12.5 Hz, ± 20 KN, and 5%, respectively.

#### 4.1.2 Comparison of mechanical feet

**Fig 5:**
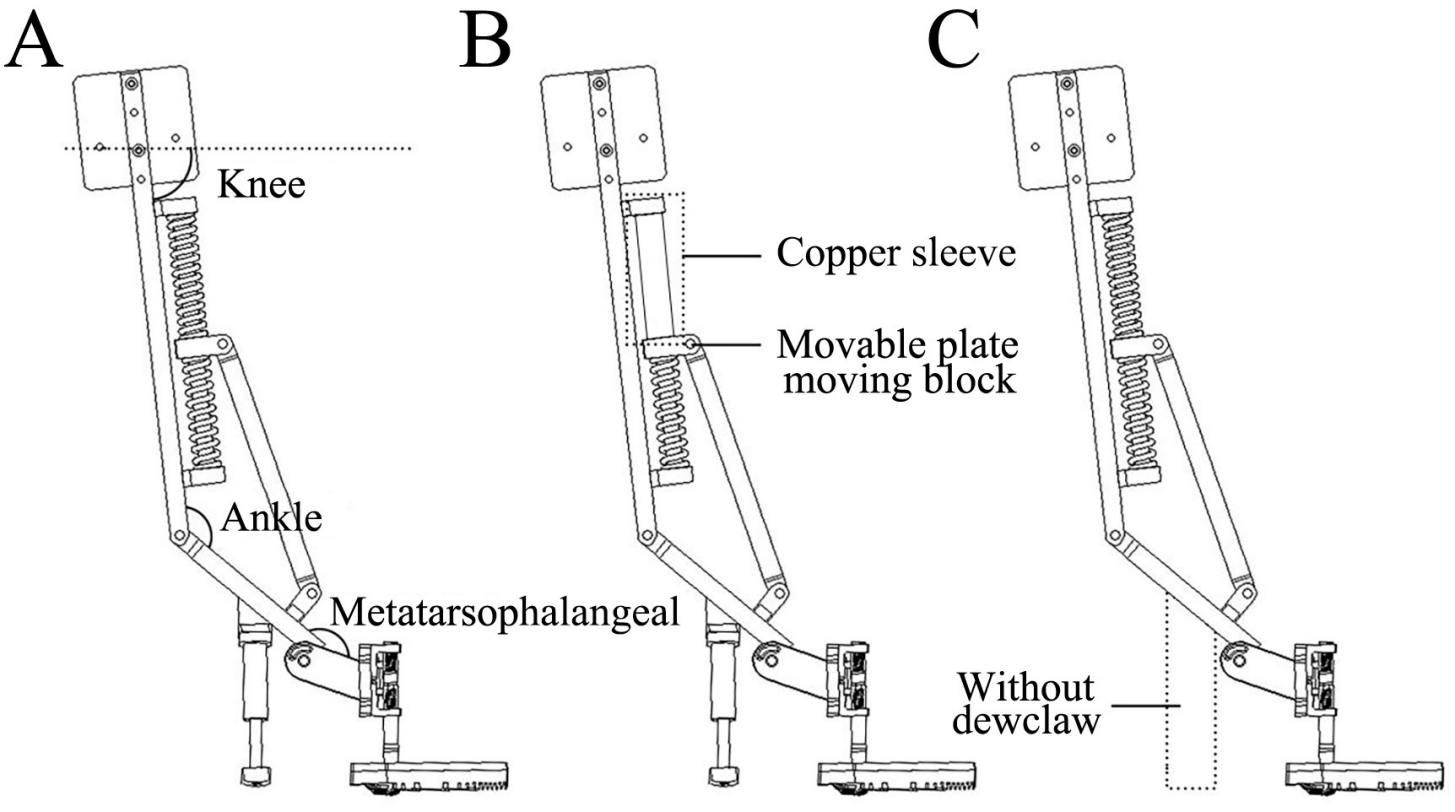
Comparison of structures of mechanical feet I, II, and III (A, B, C respectively).

To analyze the energy-saving and load-bearing performances of the bionic mechanical foot (mechanical foot I), we set up two comparative feet, including mechanical foot II with a copper sleeve added to the sliding rail to restrict the sliding of the movable plate moving block, and mechanical foot III without dewclaws (Fig 5). Based on plantar pressure and mechanical foot kinematics, we calculated the power of mechanical feet I and II, and comparatively analyzed the energy-saving performance of mechanical foot I. The plantar pressures of mechanical feet I and III during movement were compared, and the load-bearing capacity of mechanical foot I was comparatively analyzed. To examine the interaction between mechanical foot I and ice or frozen ground, we self-made some compacted frozen soil, water ice lunar soil, and ice surface as the experimental contact surfaces. The horizontal drag system was set with two levels: low speed (80 mm/s) and high speed (180 mm/s). At least three tests were conducted under each working condition.

#### 4.1.3 Data processing

The kinematic videos of the mechanical feet were processed using Phantom MultiCam (VRI) to clarify joint angle changes in the knee joint and MTP joint under various working conditions during the stance phase. The foot motion mechanics platform and the 3D force platform were used to collect the 3D forces of the junctions and the plantar pressures of the mecahnical feet during the stance phase, respectively. The stance phase was converted into a dimensionless percentile system, and then the mechanical foot functions of energy saving, load bearing and attachment mechanism on ice and frozen ground were analyzed with kinematics and mechanics.

### 4.2 Kinematics analysis of the biomimetic mechanical foot

The influence of foot structure on the two angles of the knee joint and MTP joint is shown in Table 1. Regardless of high or low speed, the ROMs of the knee joint and MTP joint in mechanical foot I are about 50 and 25 ° respectively. The ROMs of the knee joints in feet II and III are about 50 and 45 °, respectively, and the ROMs of the MTP joints are roughly 35° and 13°, respectively. In contrast to foot I, foot II has a similar ROM in the knee joint, but a larger ROM in the MTP joint. This is because the copper sleeve added on the slide rail of mechanical foot II restricts the sliding of the movable block during movement, and thereby limits the angle change of the ankle joint and increases the elastic deformation of the MTP joint. In comparison with mechanical foot I, the ROMs of the knee joint and MTP joint in mechanical foot III are smaller, which is mainly because the reduced bearing capacity of the foot without dewclaws results in a decrease in the ROM of the foot joint. Velocity does not affect the ROM of knee joint or MTP joint angle, but affects the speeds of compression and rebound of the vertical spring, thereby affecting the changes in joint angular velocity.

**Table 1:** The joint angles of the different mechanical feet.

	Movement	Touch-down		Lift-off	
		Knee joints (°)	MTP joints (°)	Knee joints (°)	MTP joints (°)
Mechanical Foot I	Low Speed	101.99±3.97	153.68±6.04	51.64±0.79	128.22±4.94
	High Speed	102.52±0.77	153.39±2.30	51.19±1.01	129.16±3.70
Mechanical Foot II	Low Speed	101.20±2.89	154.65±5.67	50.14±0.50	120.91±4.43
	High Speed	99.71±2.85	162.38±6.78	49.96±0.97	122.69±2.03
Mechanical Foot III	Low Speed	94.36±2.52	136.70±3.32	49.67±0.71	123.58±0.93
	High Speed	95.34±0.54	137.74±3.56	49.32±2.09	124.03±2.83

### 4.3 Energy-saving analysis of the biomimetic mechanical foot

To analyze energy efficiency, we measured the plantar pressures in the X, Y, and Z directions of mechanical feet I and II during the stance phase. In combination with kinematic analysis, the moment and power at the foot joint were calculated (Fig 4).

#### 4.3.1 Energy-saving analysis at low speed

**Fig 6:**
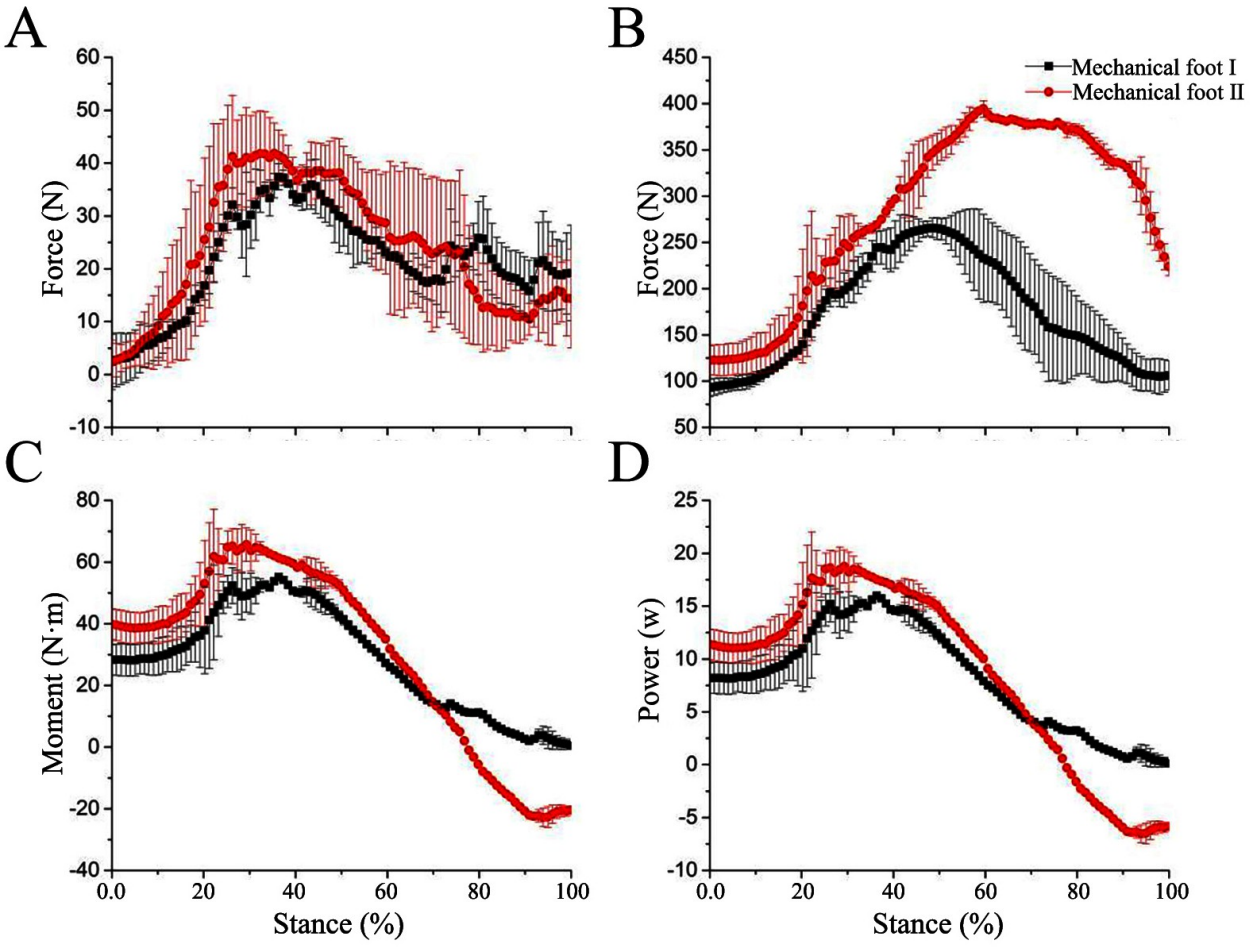
Force, moment, and power of mechanical foot at low speeds. (A) X direction force, (B) Z direction force, (C) Moment, (D) Power.

The plantar pressures in the X- and Z-direction forces of mechanical feet I and II are revealed in Fig 6. For the X-direction force, the force exerted by mechanical foot I during movement first increases and then decreases. In the later stance phase, the force exerted by mechanical foot I on the force platform gradually shifts to the horizontal drag system. Therefore, the X-direction force of mechanical foot I decreases. The force curves of mechanical feet I and II in the X direction are similar, indicating the movement of the restricted movable block little affects the X-direction force of the mechanical feet (Fig 6A). In contrast to the X-direction force, the Z-direction force is dominant, and the force platform bears a mechanical weight about 100 N on touch-down moment. As the mechanical foot moves, the support force from the force platform begins to increase, causing the Z-direction force to increase too. During the stance phase of about 50% to 100%, the support force provided by the force platform gradually decreases. The Z-direction force on mechanical foot II is larger than that of mechanical foot I. This is because the limited sliding of the movable block restricts the angle change of the ankle joint, thereby weakening the elastic energy storage and release function of the ankle joint. Therefore, the force on the sole of mechanical foot II is greater than that of mechanical foot I (Fig 6B).

At low speed, the curve between the plantar pressure in Z-direction force of mechanical foot I and the stance phase satisfies the following formula:

$$y=54.20+717.57x-712.64x^{2}\;\left({\text{R}}^{2}=86\%\right)$$

where x is the stance phase; y is the Z-direction force (N) (the meanings of x and y in the following fitting formulas are the same).

At low speed, the curve between the plantar pressure in Z-direction force of mechanical foot II and the stance phase satisfies the following formula:

$$y=46.76+948.65x-707.62x^{2}\;\left({\text{R}}^{2}=92\%\right)$$

The required driving moment for each mechanical foot increases in the 0% to 40% stance phase, but decreases in the 40% to 80% stance phase. The moment ranges of mechanical feet I and II are about 0.46–55.16 and -22.86–65.63 N·m, respectively (Fig 6C). The trend of the power curve is similar to that of the moment curve (Fig 6D). At roughly from the 0% to 80% stance phase, the motor performs positive work on the tested mechanical foot at the connection point, driving the foot to move forward. The power ranges of mechanical feet I and II are from 0.13 to 16.00 W and from -6.53 to -18.75 W, respectively. After the sliding of the movable block is limited, the power acting on mechanical foot II is enlarged. The reason is that the elastic component at the ankle joint of mechanical foot I can store elastic energy, thus reducing power consumption.

#### 4.3.2 Energy-saving analysis at high speed

**Fig 7:**
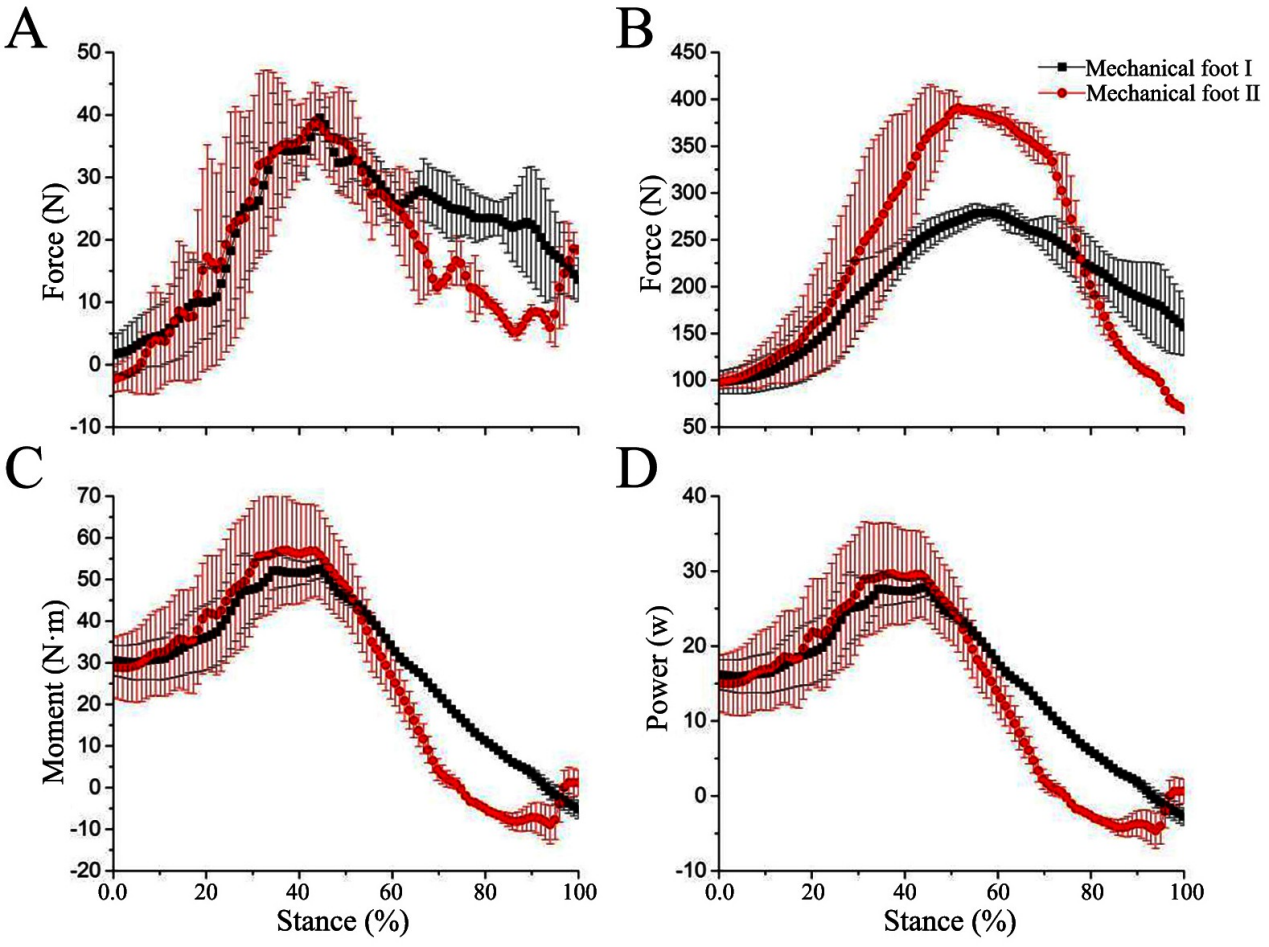
Force, moment, and power of mechanical foot at high speeds. (A) X direction force, (B) Z direction force, (C) Moment, (D) Power.

The trends of the 3D force variation curves of the mechanical feet at high speed are similar to those at low speed, but there are differences in the magnitude in the curves. At high speed, the tested mechanical foot experiences higher pressure on the sole compared to the situation at low speed (Fig 7). Specifically, at high speed, the difference between mechanical feet I and II in the X direction is small (Fig 7A), while the numerical difference in the Z-direction force is significant (Fig 7B). The peak Z-direction force of mechanical foot II increases by about 60% in contrast to mechanical foot I.

At high speed, the curve between the plantar pressure in Z-direction force of mechanical foot I and the stance phase satisfies the following formula:

$$y=46.47+713.81x-605.17x^{2}\;\left({\text{R}}^{2}=92\%\right)$$

Simultaneously, the curve between the plantar pressure in Z-direction force of mechanical foot II and the stance phase at high speed satisfies the following formula:

$$y=\text{-}6.30+1358.57x-1316.77x^{2}\;\left({\text{R}}^{2}=85\%\right)$$

During high-speed movement, the moment of the junction of mechanical feet increases in the 0%—40% stance phase, and decreases in the 40%—80% stance phase (Fig 7C). The moment ranges of mechanical feet I and II are from about -5.22 to -52.54 N·m and -8.91 to -56.99 N·m, respectively, and the power ranges are from about -2.77 to -27.85 W and -4.63 to -29.63 W, respectively (Fig 7D). At low and high speeds, the maximum power of mechanical foot II is about 1.17 and 1.06 times that of mechanical foot I respectively. The power variation ranges of mechanical feet II and I at high speed are relatively close to those at low speed. This is because although mechanical foot II limits the energy storage function of the elastic components at the ankle joint, this function at other parts (foot ends) of mechanical foot II is strengthened and compensated at high speed, which reduces the difference in power between mechanical feet I and II in high-speed motion.

### 4.4 Loading-bearing analysis of the biomimetic mechanical foot

#### 4.4.1 Loading-bearing analysis at low speed

**Fig 8:**
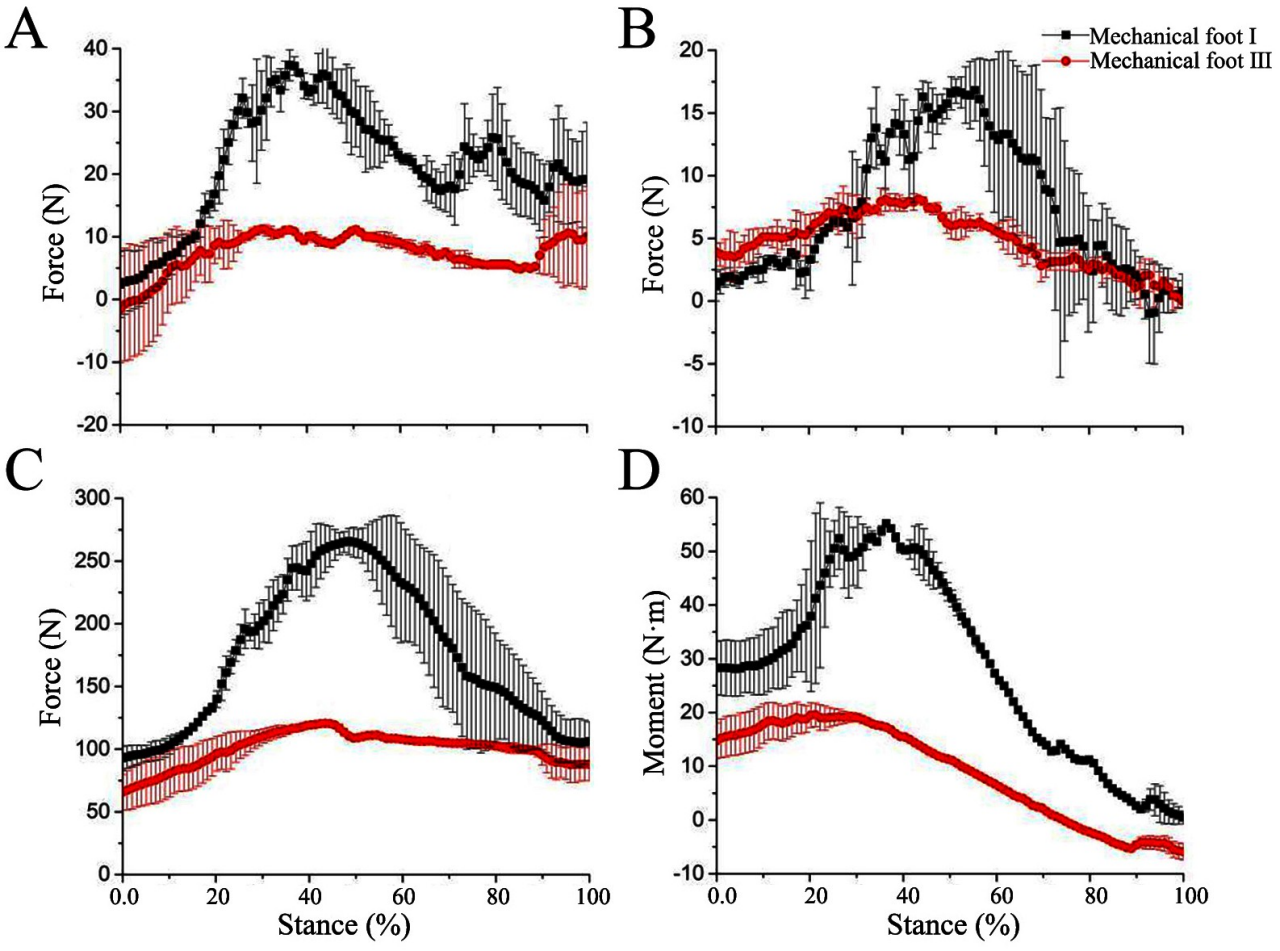
3D force and moment at low speed. (A) X-direction force, (B) Y-direction force, (C) Z- direction force, (D) Moment.

Mechanical foot III exhibits a smaller traction force (X-direction force) compared to mechanical foot I (Fig 8A). However, the range of variation in Y-direction force for mechanical foot I is larger than that of mechanical foot III. This result suggests that the dewclaws on mechanical foot I enhance lateral force and help prevent lateral sliding (Fig 8B). The variation range of Z-direction ground reaction force (GRF) in mechanical foot I is larger than that of mechanical foot III. Specifically, the maximum GRFs of mechanical feet I and III are about 275 and 125 N respectively. This result indicates the ground provides foot I with sufficient support to complete the touch-down motion (Fig 8C). The maximum moment of mechanical feet I and III is about 55 and 20 N·m, respectively (Fig 8D). Mechanical foot I can transmit force to the spring through dewclaws, converting excessive energy into elastic energy for storing and sharing the force at the load-bearing toe. Hence, in low-speed, foot I improves its load-bearing ability through the action of dewclaws.

At low speed, the curve between the plantar pressure in Z-direction force of mechanical foot III and the stance phase satisfies the following formula:

$$y=67.07+173.34x-160.09x^{2}\;\left({\text{R}}^{2}=89\%\right)$$

#### 4.4.2 Loading-bearing analysis at high speed

**Fig 9:**
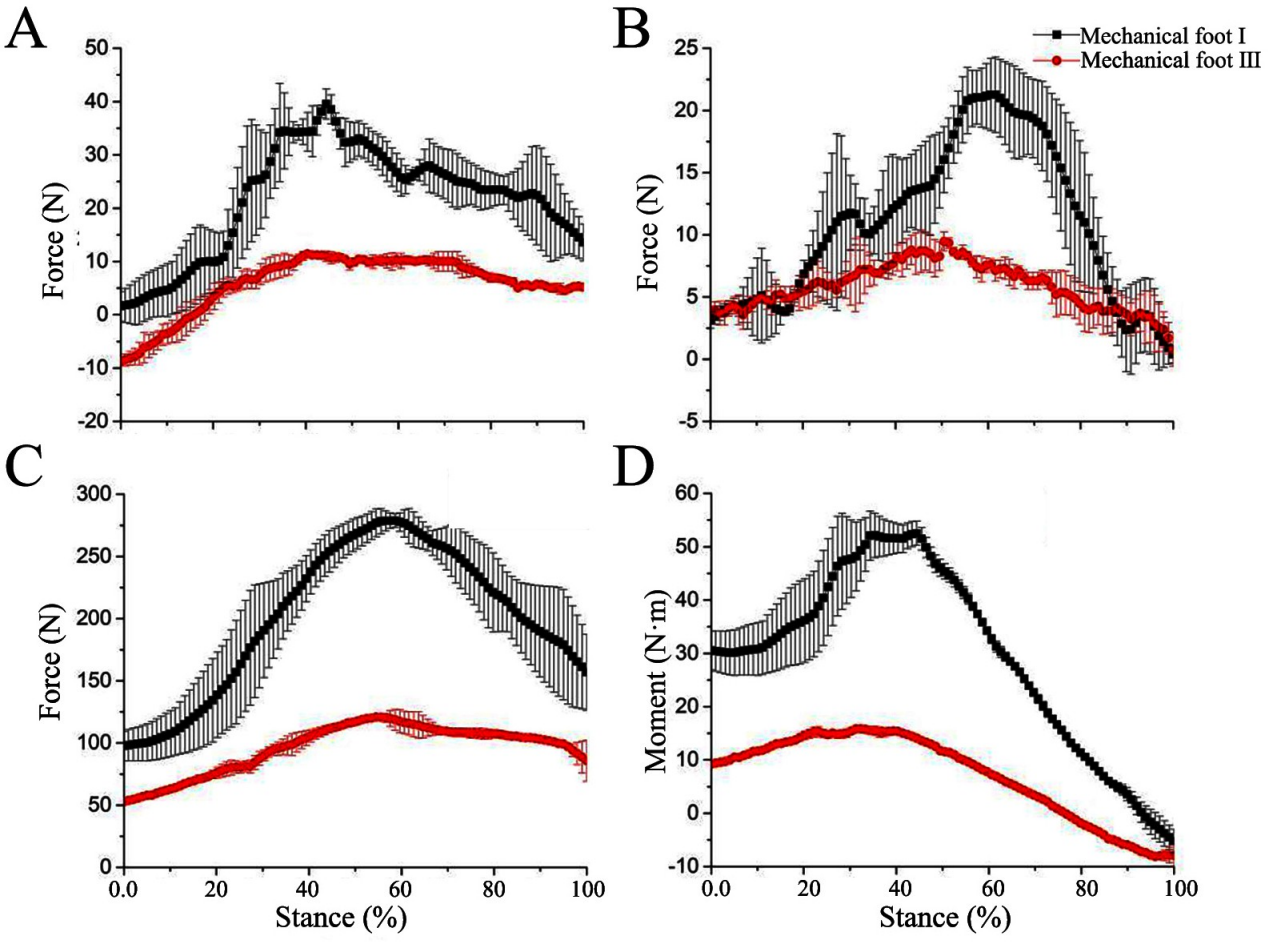
3D force and moment at high speed. (A) X-direction force, (B) Y-direction force, (C) Z- direction force, (D) Moment.

The 3D forces and moment of the mechanical feet at high speed are similar to those at low speed, except for some differences in the range and magnitude of the changes. In comparison with the results at low speed, the peak X-, Y-, and Z-direction forces of mechanical foot III at high speed are 1.02, 1.17 and 1.01 times those at low speed, respectively (Fig 9A–9C). In contrast to mechanical foot III, the peak X-, Y-, and Z-direction forces of mechanical foot I at high speed increase by about 2.54, 1.25 and 1.31 times, respectively, and the moment rises by about 2.58 times (Fig 9D). In high-speed, the dewclaws significantly improve the load-bearing performance of the entire mechanical foot.

At high speed, the curve between the plantar pressure in Z-direction force of mechanical foot III and the stance phase satisfies the following formula:

$$y=43.05+223.02x-175.63x^{2}\;\left({\text{R}}^{2}=95\%\right)$$

### 4.5 Attachment mechanism of the biomimetic mechanical foot on ice and frozen ground

During the interaction between the foot and ice or frozen ground, the foot motion mechanics platform collected the 3D forces at the junction of the mechanical foot and obtained kinematic data. The X-, Y- and Z-direction forces are the traction force, lateral force and squeezing force of the mechanical foot, respectively. The movement process of the biomimetic mechanical foot is divided into touch-down, mid-down, and lift-off at the 0%, 50%, and 100% of stance phase respectively.

#### 4.5.1 Mechanism of interaction with frozen ground

**Fig 10:**
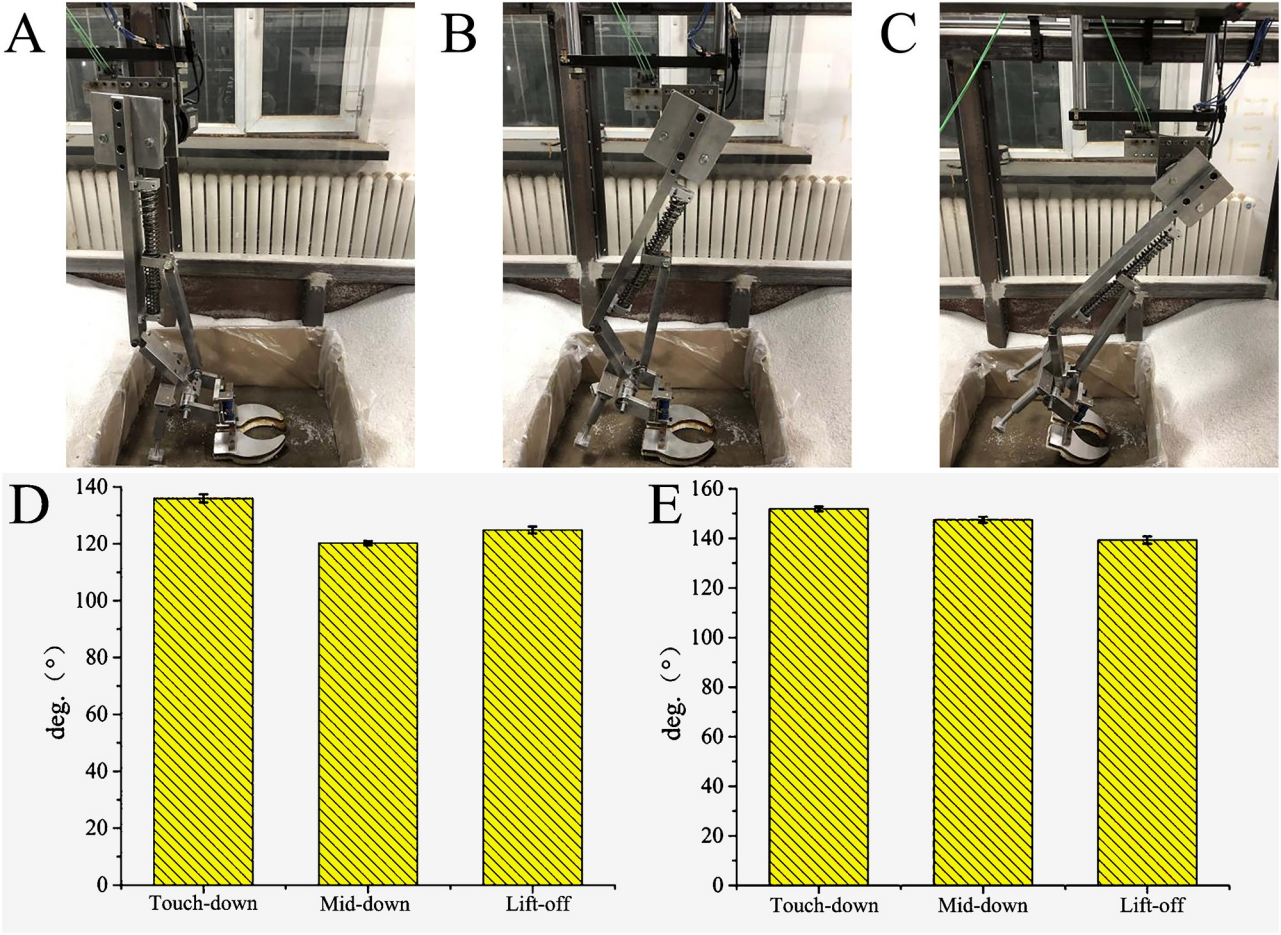
Motion process and joint angles of biomimetic mechanical foot on frozen ground. (A) Touch-down, (B) Mid-down, (C) Lift-off, (D) Ankle joint angle, (E) MTP joint angle.

The ankle joint angles of the tested mechanical foot are about 134.17, 121.00, and 121.07 ° at the touch-down, mid-down, and lift-off, respectively, and the MTP joint angles are about 147.07, 140.83 and 139.90 °, respectively. When the mechanical foot moved on frozen ground, the ROMs of the ankle joint and the MTP joint are about 13.17 and 7.17°, respectively (Fig 10).

**Fig 11:**
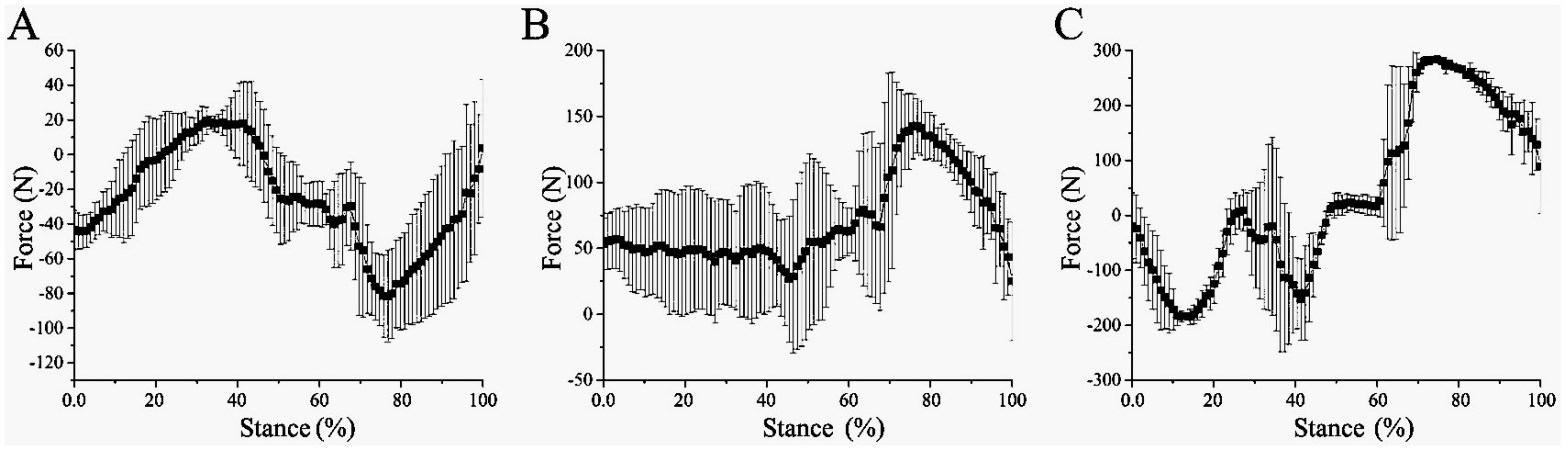
3D force between the mechanical foot and frozen ground. (A) X-direction force, (B) Y- direction force, (C) Z-direction force.

The X-, Y- and Z-direction forces when the biomimetic mechanical foot interacts with frozen soil are shown in Fig 11. Generally, the Z-direction force initially decreases, then increases, and finally declines again. In the early stance phase, the weight of the biomimetic mechanical foot is primarily supported by the ground. As the mechanical foot moves, the GRF in the Z-direction increases, causing the Z-direction force to decrease. Subsequently, as the mechanical foot lifts off the ground, its center of mass shifts forward and gradually transfers onto the frame, resulting in an increase in the Z-direction force. Finally, just before liftoff, the toe contacts the ground to support the mechanical foot, causing the Z-direction force to decrease once again. The peak X-, Y- and Z-direction forces of the bionic mechanical foot occur at 32.32%—76.77%, 43.32%—76.77%, and 14.14%—77.78% of the stance phase respectively. During the movement, the X-, Y- and Z-directions forces of the bionic mechanical foot vary by about 101.07, 118.00 and 469.19 N, respectively.

On the frozen ground, the curve between the Z-direction force at the junction of the mechanical foot and the stance phase satisfies the following formula:

$$y=\text{-}31.67\text{-}1270.27x+4124.71x^{2}\text{-}2677.38x^{3}\;\left({\text{R}}^{2}=84\%\right)$$

where x is the stance phase; y is the Z-direction force (N) (the meanings of x and y in the following fitting formulas are the same).

#### 4.5.2 Mechanism of interaction with water ice lunar soil

**Fig 12:**
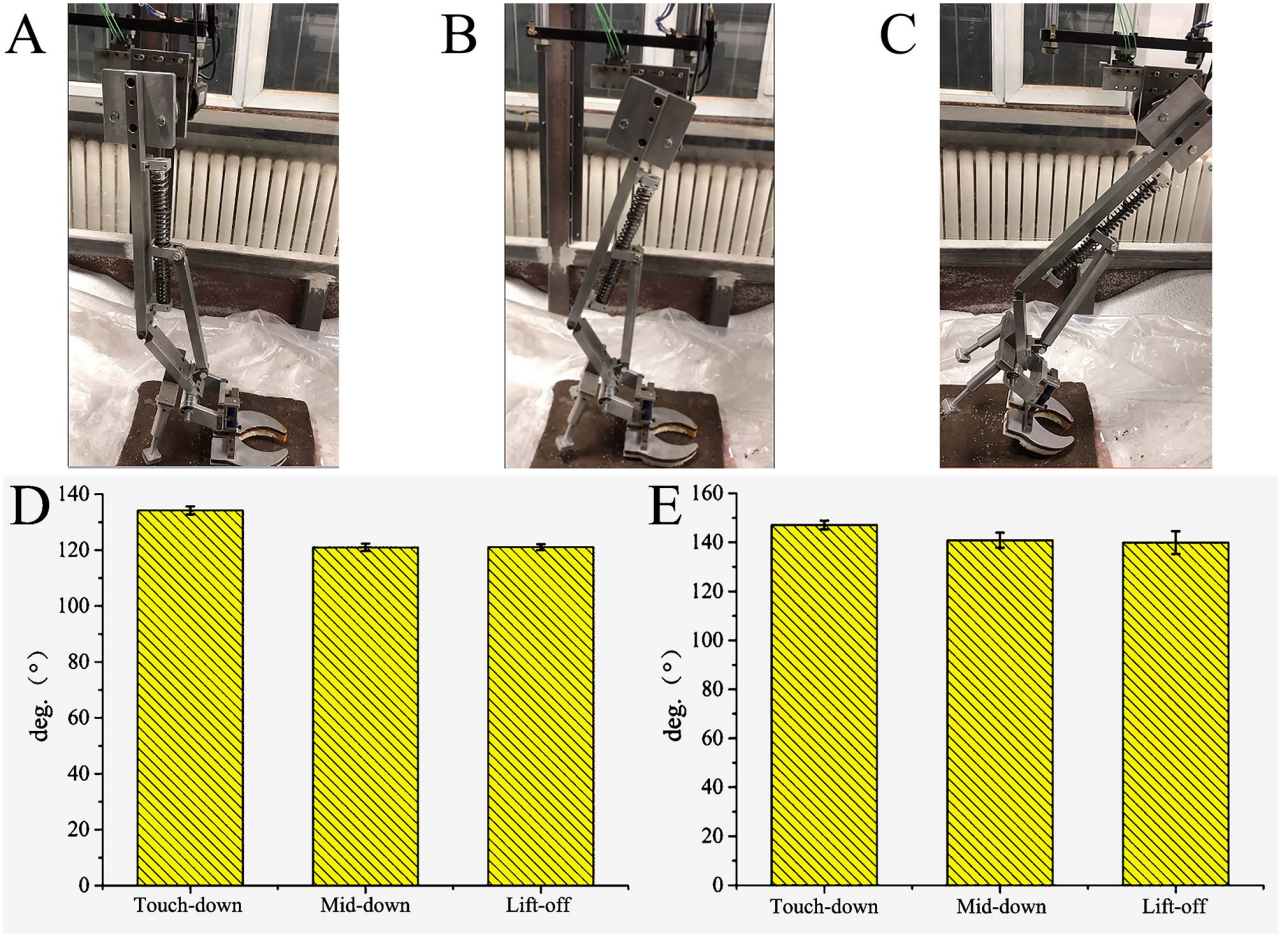
Motion process and joint angles of biomimetic mechanical foot on water ice lunar soil. (A) Touch-down, (B) Mid-down, (C) Lift-off, (D) Ankle joint angle, (E) MTP joint angle.

The ankle joint angles of the mechanical foot are about 135.93, 120.23, and 124.83° at the touch-down, mid-down, and lift-off, respectively, and the MTP joint angles are about 151.90, 147.50, and 139.30°, respectively. When the mechanical foot moves on water ice lunar soil, the ROMs of the ankle joint and the MTP joint are about 15.70° and 12.60°, respectively (Fig 12).

**Fig 13:**
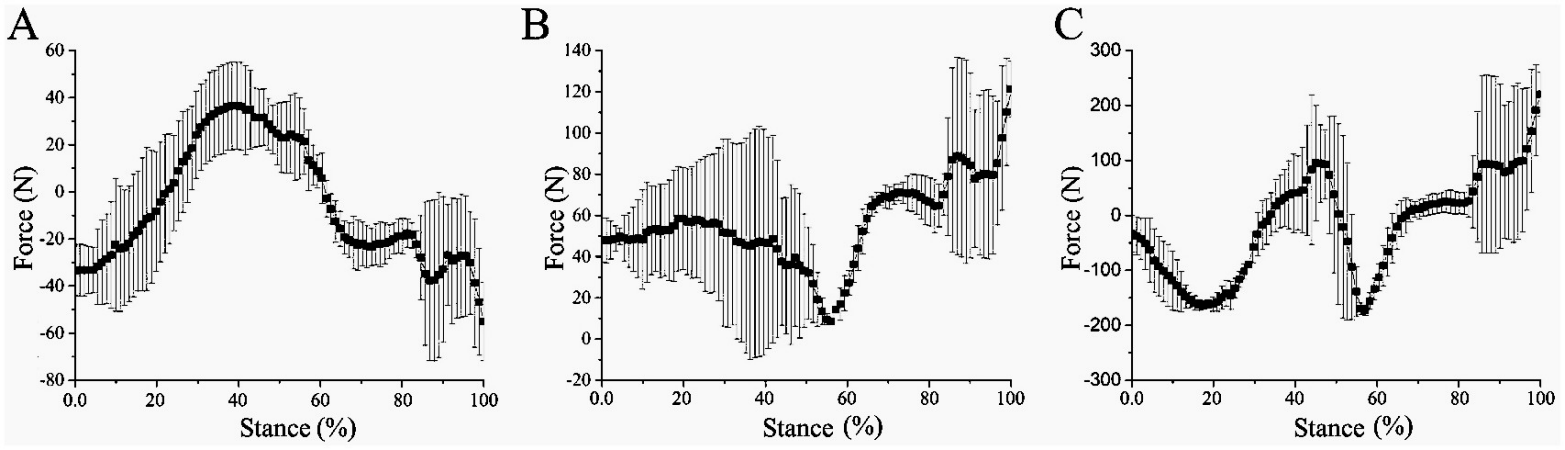
3D force between the mechanical foot and water ice lunar soil. (A) X-direction force, (B) Y-direction force, (C) Z-direction force.

The 3D forces in the X, Y and Z directions when the biomimetic mechanical foot interacts with water ice lunar soil are shown in Fig 13. The peak X-, Y- and Z-direction forces of the foot occur at 39.56%, 54.95% and 57.14% of the stance phase, respectively. In comparison with frozen ground, the peak force time of the mechanical foot in water ice lunar soil shifts rightward. This is because at the same speed, the mechanical foot in the later stance phase can still operate normally on the frozen soil when it slips during the water ice lunar soil movement, reducing the contact time with the water ice lunar soil. During the movement, the X-, Y- and Z-direction forces of the bionic mechanical foot vary by about 91.61, 112.67, and 392.33 N, respectively. In contrast to frozen ground, the variation range in 3D forces of the mechanical foot on water ice lunar soil is smaller. When water ice lunar soil interacts with the mechanical feet, the force at the junction of the foot is relatively small, which strengthens the force between the foot bottom and water ice lunar soil, thereby improving the attachment performance of the foot.

On water ice lunar soil, the curve between the Z-direction force at the junction of mechanical foot and the stance phase satisfies the following formula:

$$y=\text{-}123.83+357.28x\text{-}658.75x^{2}+581.03x^{3}\;\left({\text{R}}^{2}=51\%\right)$$

#### 4.5.3 Mechanism of interaction with ice surface

**Fig 14:**
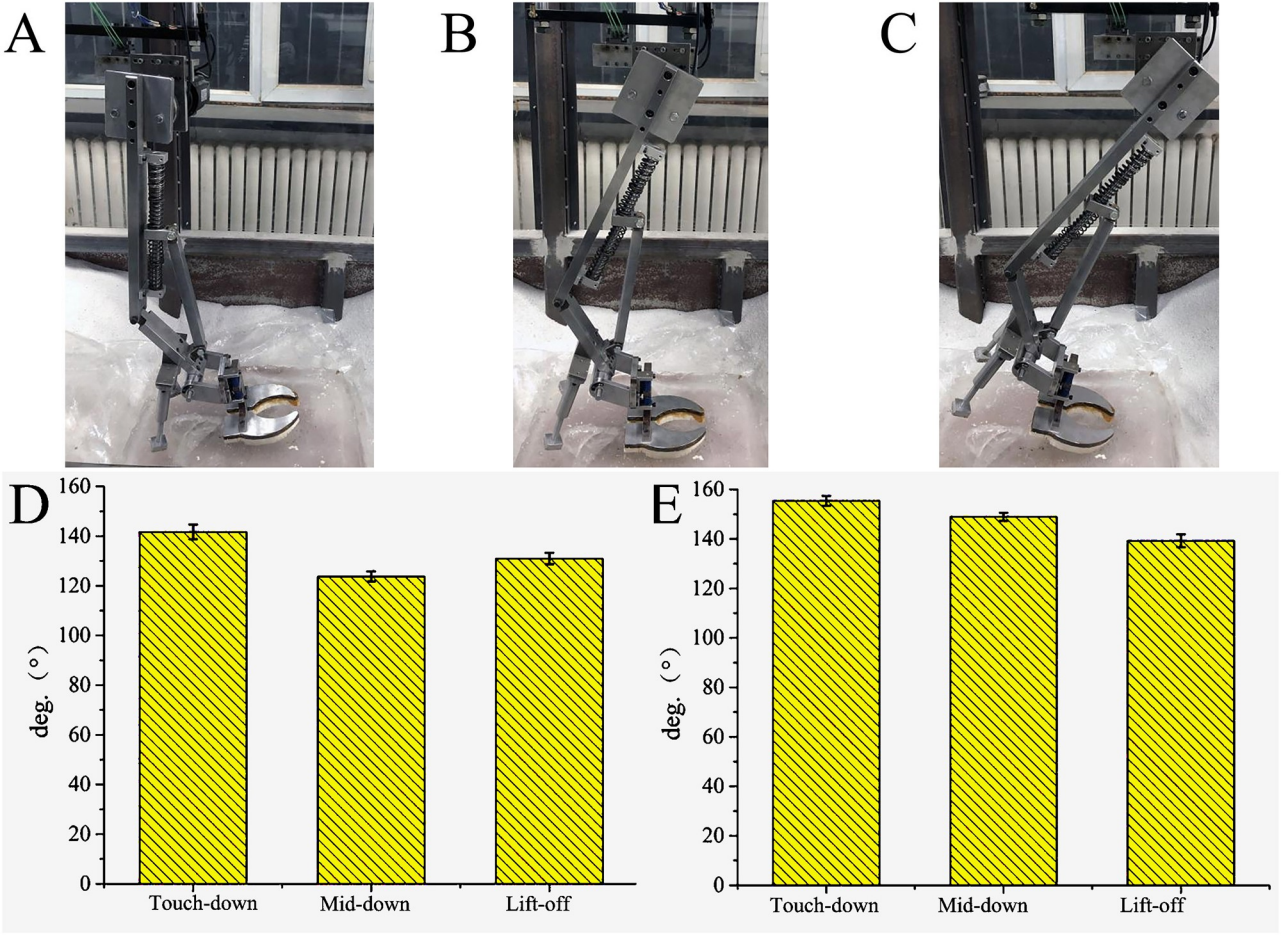
Motion process and joint angles of biomimetic mechanical foot on ice surface. (A) Touch-down, (B) Mid-down, (C) Lift-off, (D) Ankle joint angle, (E) MTP joint angle.

The ankle joint angles of the tested mechanical foot at the touch-down, mid-down, and lift-off are about 141.70, 123.77 and 130.93 °, respectively, and the MTP joint angles are about 155.40, 148.93 and 139.30 °, respectively. When the mechanical foot moves on the ice, the ROMs of the ankle joint and the MTP joint are about 17.93° and 16.10°, respectively (Fig 14).

**Fig 15:**
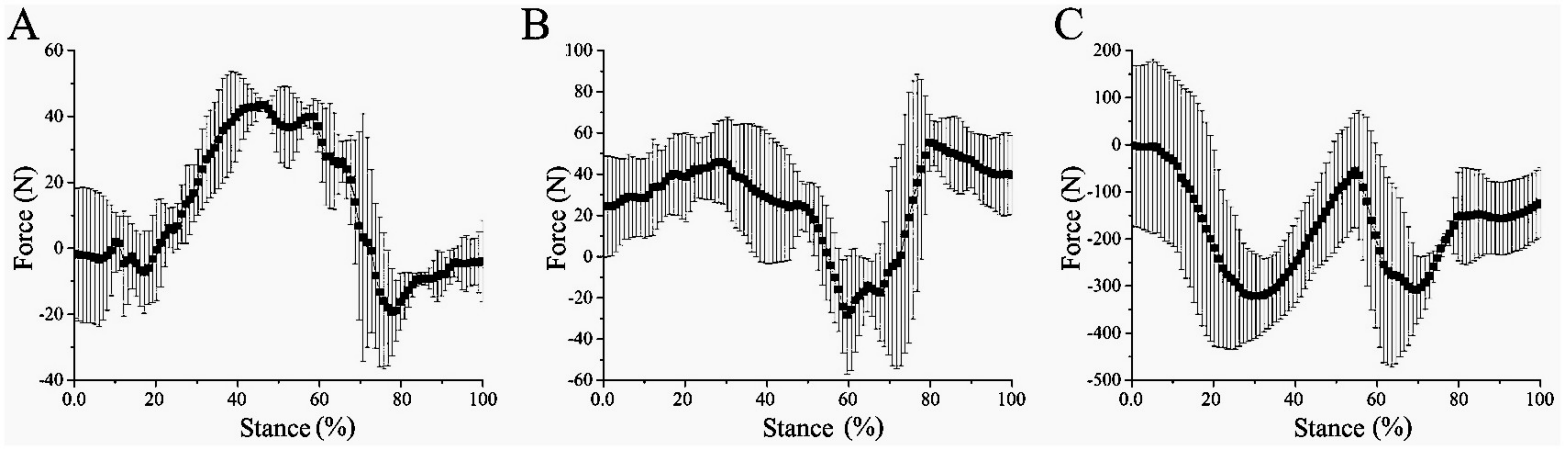
3D force between the mechanical foot and ice surface. (A) X-direction force, (B) Y- direction force, (C) Z-direction force.

The 3D forces in the X, Y and Z directions when the biomimetic mechanical foot interacts with the ice surface are revealed in Fig 15. The peak X-, Y- and Z-direction forces of the bionic mechanical foot locate at 43.43%, 59.60%, and 69.70% of the stance phase respectively. In comparison with frozen and water ice lunar soil, the peak force time of the mechanical foot on the ice surface shifts rightwards, shortening the contact time between the foot and the ice surface at the later stance phase, which is beneficial for reducing the slipping of the mechanical foot. During the movement, the X-, Y- and Z-direction forces of the bionic mechanical foot vary by about 62.96, 83.7 and 319.85 N, respectively, with the lowest variation among the three types of ground. When the biomimetic mechanical foot interacts with the smoother ice surface, its joint ROM increases, and the changes in the 3D forces at the foot joint decrease.

On the ice surface, the curve between the Z-direction force connected to the upper end of the bionic mechanical foot and the touch-down time satisfies the following formula:

$$y=42.55\text{-}1648.48x+2893.44x^{2}\text{-}1450.74x^{3}\;\left({\text{R}}^{2}=44\%\right)$$

In summary, as for the performance on frozen ground, the good load-bearing capacity of mechanical feet helps increase traction and attachment performance. Additionally, the excellent energy-saving characteristics of mechanical feet improve their motion efficiency on frozen ground, making them more adaptable to this challenging environment. These features are significant for the practical application of mechanical feet on frozen ground.

This study provides a detailed investigation into the kinematic and mechanical characteristics of a bionic mechanical foot on frozen ground, ice, and water-ice lunar soil simulant. However, there are certain limitations to consider. The experiments are carried out in a controlled laboratory environment, which may not entirely emulate the dynamic and unpredictable conditions experienced in real-world applications. While the mechanical foot is tested on three types of ground (frozen ground, ice, and lunar soil simulant), the diversity of terrains that a robotic system may face in practical scenarios is extensive. Therefore, the study may not encompass all possible variations and challenges.

## 5. Conclusions

The hind limb joint angles of reindeer exhibit remarkable adaptability, and allow reindeer to adjust to various movement gaits and speeds. We developed three mechanical feet by leveraging the kinematic traits of reindeer hooves during the stance phase. The dual performance of the biomimetic mechanical foot in energy efficiency and load-bearing capacity was affirmed on a 3D platform. The kinematics and mechanics governing the interaction between the foot and frozen ground were explored in depth on a bionic mechanical foot motion mechanics platform, and delved into the underlying mechanisms to fit mechanical formulas.

The movable block of the biomimetic mechanical foot is linked to elastic components at the ankle joint. During low-speed motion, these elastic components effectively store elastic energy, leading to a reduction in power consumption. In high-speed motion, the compensatory effect of multiple elastic components within the mechanical foot ensures that the movable plate or the moving block minimally influences the power of the mechanical foot. The bionic mechanical foot transmits force to its upper end spring through dewclaws. This process converts any surplus energy into elastic energy for storing and sharing the load on the weight-bearing toe, and subsequently enhances the load-bearing capacity of the mechanical foot.

The mechanical foot exhibits the smallest ROM at the ankle and MTP joints on frozen, followed by water ice lunar soil, and the largest ROM on ice surface. An increased ROM implies the biomimetic mechanical foot needs to adjust its motion posture and change joint angles to maintain stable motion on ice surfaces and obtain sufficient ground force. When the biomimetic mechanical foot engages with ice and frozen ground, its motion and mechanical patterns differ, likely contributing to motion stability and slip reduction. The load-bearing capacity of the mechanical foot is pivotal in enhancing X-direction traction and attachment performance upon interaction with ice and frozen ground. The energy-saving capacity of the biomimetic mechanical foot improves movement efficiency and its adaptability to traverse ice and frozen ground.
